# Complete Atrial-Specific Knockout of Sodium-Calcium Exchange Eliminates Sinoatrial Node Pacemaker Activity

**DOI:** 10.1371/journal.pone.0081633

**Published:** 2013-11-21

**Authors:** Sabine Groenke, Eric D. Larson, Sarah Alber, Rui Zhang, Scott T. Lamp, Xiaoyan Ren, Haruko Nakano, Maria C. Jordan, Hrayr S. Karagueuzian, Kenneth P. Roos, Atsushi Nakano, Catherine Proenza, Kenneth D. Philipson, Joshua I. Goldhaber

**Affiliations:** 1 Cardiovascular Research Laboratories, David Geffen School of Medicine, University of California Los Angeles, Los Angeles, California, United States of America; 2 Department of Physiology & Biophysics, University of Colorado School of Medicine, Denver, Colorado, United States of America; 3 Heart Institute, Cedars-Sinai Medical Center, Los Angeles, California, United States of America; 4 Department of Molecular, Cell and Developmental Biology, Eli and Edythe Broad Center of Regenerative Medicine and Stem Cell Research, University of California Los Angeles, Los Angeles, California, United States of America; University of Naples Federico II, Italy

## Abstract

The origin of sinoatrial node (SAN) pacemaker activity in the heart is controversial. The leading candidates are diastolic depolarization by “funny” current (I_f_) through HCN4 channels (the “Membrane Clock“ hypothesis), depolarization by cardiac Na-Ca exchange (NCX1) in response to intracellular Ca cycling (the "Calcium Clock" hypothesis), and a combination of the two (“Coupled Clock”). To address this controversy, we used Cre/loxP technology to generate atrial-specific NCX1 KO mice. NCX1 protein was undetectable in KO atrial tissue, including the SAN. Surface ECG and intracardiac electrograms showed no atrial depolarization and a slow junctional escape rhythm in KO that responded appropriately to β-adrenergic and muscarinic stimulation. Although KO atria were quiescent they could be stimulated by external pacing suggesting that electrical coupling between cells remained intact. Despite normal electrophysiological properties of I_f_ in isolated patch clamped KO SAN cells, pacemaker activity was absent. Recurring Ca sparks were present in all KO SAN cells, suggesting that Ca cycling persists but is uncoupled from the sarcolemma. We conclude that NCX1 is required for normal pacemaker activity in murine SAN.

## Introduction

Sinus node disease is associated with death from severe bradycardia. It is also associated with a high incidence of supraventricular tachycardia and accounts for approximately half of the 370,000 pacemakers implanted in the United States in 2010 at an average cost of $65,538 and totaling $24B [1]. However, the mechanism underlying spontaneous pacemaker activity in the sinoatrial node (SAN) is uncertain. Two competing hypotheses dominate the field: the "Membrane Clock" (M clock) hypothesis that emphasizes the role of “funny” current (I_f_) through HCN4 channels in the generation of pacemaker activity, and the "Calcium Clock" (Ca clock) hypothesis that focuses on the role of spontaneous Ca release from the sarcoplasmic reticulum (SR). A third hypothesis, known as the “Coupled Clock,” attempts to combine key elements of the first two. In the M clock model, I_f_ current activates when the SAN cell repolarizes to its resting membrane potential. Inward I_f_ depolarizes the cell in diastole until the threshold is reached for activation of the L-type Ca current (I_Ca_), which then triggers an action potential (AP). An appealing aspect of this hypothesis is that AP firing rate seems to correlate with changes in I_f_ produced by sympathetic (β-adrenergic) and parasympathetic (muscarinic) agonists and antagonists [2]. Clinically, the response of heart rate in patients to I_f_-specific drugs parallels cellular studies, supporting the relevance of I_f_ and the M clock to pacemaker activity.

However, a competing hypothesis has emerged during the past decade: the Ca clock hypothesis suggests that pacemaking is dependent upon periodic Ca transients [3], which are also modulated by the β-adrenergic system [4]. Proponents of the Ca clock hypothesis have shown that the SR spontaneously generates rhythmic Ca release events whose frequency depends upon 1) SR refilling rate in response to Ca ATPase (SERCA) activity and 2) ryanodine receptor (RyR) recovery from inactivation following depolarization [5], [6]. Rhythmic Ca release is then “coupled” to the surface membrane via Ca-dependent regulation of sarcolemmal ion channels and transporters, thus enabling the Ca-clock to drive SAN APs [4]. The electrogenic Na-Ca exchanger (NCX) in particular is postulated to play a critical role in coupling intracellular Ca release to membrane depolarization by accelerating late diastolic depolarization of the surface membrane in response to local Ca release (LCR) from the SR. Evidence in favor of the pivotal role of NCX is that low-sodium bath solutions (which prevent NCX from generating an inward current) inhibit spontaneous APs in isolated guinea pig SAN cells [7]. Depletion of SR Ca with ryanodine also perturbs pacemaker activity in rabbit SAN cells [8]. However, both of these manipulations could also alter SAN activity through unexpected changes in I_f_ and I_Ca_. Genetic approaches using inducible knockouts of NCX have mostly supported the role of the exchanger in modulating pacemaker activity. Yet none of these models has completely eliminated SAN NCX activity [9], [10]. We have overcome these limitations by producing atrial-specific NCX1 KO mice where NCX1, the exclusive isoform of NCX found in cardiac sarcolemma [11], is 100% ablated from all atrial myocytes including SAN cells. These mice allow, for the first time, investigation of SAN activity in the complete absence of NCX1. Our results support the hypothesis that NCX1 is indeed required for pacemaker activity of SAN cells.

## Results

### Knockout of NCX1 in the atrium and sinoatrial node

**Figure 1:**
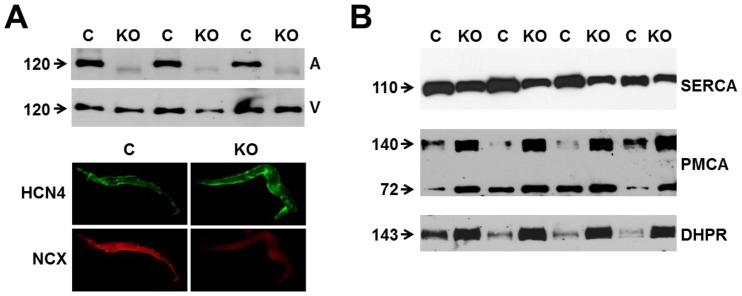
Expression of NCX1 and other Ca handling proteins in atrial-specific NCX1 knockout mice. **A**. Immunoblot (top) of mouse atrial (A) and ventricular (V) homogenate using an antibody to NCX1 in wildtype control (C) and atrial-specific NCX1 KO mice. There is complete absence of NCX1 protein in the immunoblot. The new band in the KO lanes at ∼110 kDa represents nonfunctional NCX in KO atria after excision of exon 11 by Cre recombinase [13]. Ventricular expression of NCX1 is unaffected in atrial-specific KO mice. The bottom panel shows immunofluorescence of isolated SAN node myocytes from control (C) and KO hearts. Myocytes were co-immunolabled with antibodies against HCN4 and NCX1. Both control and KO SAN cells stained positive for HCN4, but only control SAN cells showed staining of NCX at the membrane. **B**. Immunoblots of sarcoendoplasmic reticulum Ca ATPase (SERCA), plasma membrane Ca pump (PMCA; the lower band at 72 kDa represents an active proteolytic fragment), and dihydropyridine receptor (DHPR) in control (C) and NCX1 KO atria. Note the reduction in SERCA and the increase in PMCA and DHPR in KO.

To achieve complete deletion of NCX1 in SAN cells, we created atrial-specific NCX1 KO mice using a Cre/loxP system with expression of Cre under the control of the endogenous sarcolipin (SLN) promoter. In heart, SLN is expressed exclusively in the atrium, including the SAN [12], and SLN Cre heterozygous mice have no cardiac phenotype including electrocardiographic abnormalities (data not shown). We mated SLN Cre mice with our previously described NCX1 exon 11 floxed mice (NCX1^fx/fx^) [13] to produce atrial-specific NCX1 KO mice. NCX1^fx/fx^ littermates served as control (referred to as WT) for all experiments. KO mice survived into adulthood despite the complete absence of NCX1 in the atrium as measured directly by immunoblots from atrial homogenates probed with a well-characterized NCX1 antibody (Fig. 1A). The faint lower MW band appearing in the KO lanes represents nonfunctional NCX after excision of exon 11 by Cre recombinase [13]. The level of NCX1 in ventricular homogenate was unaffected in the KO (Fig. 1A, upper panel). The atrial KO of NCX1 included the SAN as demonstrated by immunofluorescence from enzymatically isolated SAN cells (Fig. 1A, lower panels). Whereas SAN cells from both WT (n = 15) and KO (n = 12) mice expressed HCN4 protein, only WT cells exhibited positive NCX1 immunofluorescence. KO cells only had diffuse background staining equivalent to that obtained when the primary antibody was omitted (data not shown).

In response to the absence of NCX1, the levels of other cardiomyocyte proteins that regulate Ca could adapt to compensate. Indeed, immunoblots of atrial homogenates revealed a 3.1±0.3-fold increase in expression of the plasma membrane Ca pump (PMCA), the only alternative sarcolemmal Ca efflux mechanism to NCX (Fig. 1B; n = 8 WT, 8 KO; *P*<0.001). We also found a 72±12% *decrease* in SERCA, the major Ca reuptake mechanism for the SR (n = 8 WT, 8 KO; *P*<0.001). As described below, the reduced SERCA level does not seem to compromise SR function. Levels of dihydropyridine (DHPR) protein (used to measure L-type Ca channel expression) increased by a factor of 2.6±0.2 (Fig. 1B; n = 16 WT, 16 KO; *P*<0.001). These data contrast with our previous results in ventricular-specific NCX1 KO mice in which adaptations of Ca-regulatory ventricular protein levels did not occur [13]. However, the SERCA data are reminiscent of the response of embryonic heart tubes to global KO of NCX1; in this situation, SERCA levels were also decreased [14]. Our SERCA data also contrast with those of Herrmann et al. [9] who found that SERCA increased in a different and incomplete NCX1 KO model.

### NCX Current and SR Function

**Figure 2:**
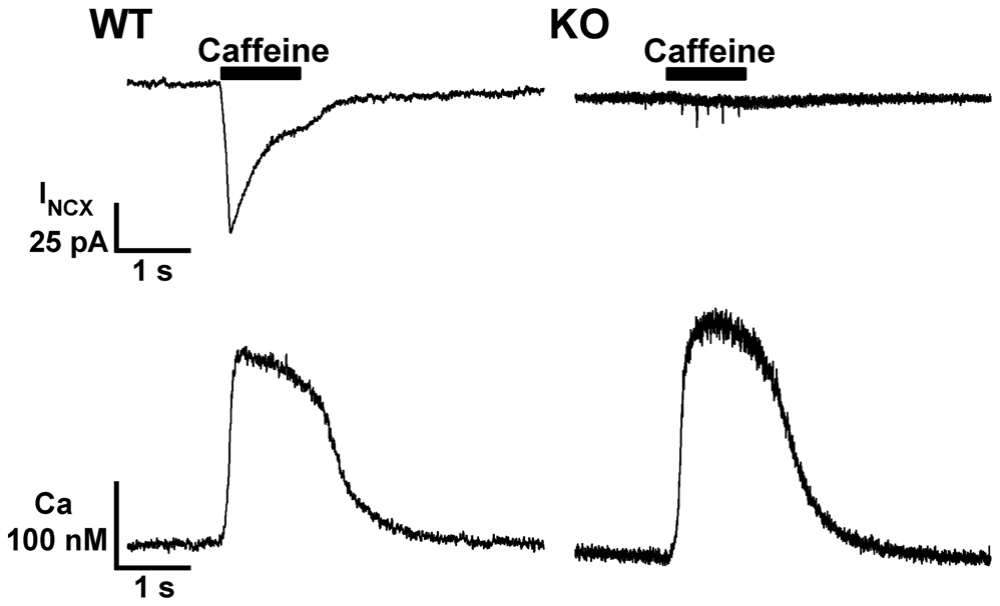
Caffeine-induced NCX currents and SR Ca load in WT and NCX1 knockout SAN cells. Membrane current (top) and Ca transients (bottom) from representative patch clamped WT (left) and KO (right) SAN cells loaded with the Ca-indicator fura-2. Cells were held at a constant voltage of -40 mV. A 1 s administration of bath solution containing 5 mM caffeine (black bar, upper panels) released SR Ca stores producing similar Ca transients in both cell types (bottom panels). The caffeine-induced Ca transient always produced an inward NCX current (I_NCX_) in WT cells (top left), but never in KO (top right) despite Ca transients of normal kinetics and amplitude (see text for details).

We next used the patch clamp technique to directly measure NCX current (I_NCX_) and intracellular Ca in enzymatically isolated SAN cells loaded with the Ca indicator fura-2 AM (Fig. 2). Cells were exposed to a 1 s puff of caffeine (5 mM) using a rapid solution exchanger to release SR Ca and generate I_NCX_. [15], [16], [17]. Conditioning pulses preceded all measurements to equilibrate the SR. Caffeine-induced Ca release generated I_NCX_ in all 9 WT cells tested (Fig. 2, upper left). In contrast, we never detected I_NCX_ in response to caffeine-induced Ca release in 24 NCX1 KO cells (Fig. 2, upper right). Thus both biochemical (Fig. 1) and electrophysiological data demonstrate complete absence of NCX protein in the atria, including the SAN, of these NCX1 KO mice.

Despite the reduction in SERCA protein expression described above for NCX1 KO, we found no significant alteration in the amplitude of caffeine-induced Ca transients, which reflect SR Ca load (WT: 392±41 nM, n = 9; KO: 475±55 nM, n = 24; *P* = 0.38), and no significant difference in resting cytosolic Ca (WT: 124±17 nM, n = 9; KO: 94±16 nM, n = 24; *P* = 0.29). The rate of relaxation (τ) of the declining phase of the Ca transient (fit to a single exponential) was also similar in WT and KO (WT 375±15 ms, n = 9; KO 421±37 ms, n = 24; *P* = 0.47). Additionally, we never saw any evidence of induced Ca waves or overload in KO cells after conditioning pulses. These results indicate that KO of NCX1 (and adaptive reduction of SERCA and increase in PMCA) does not significantly alter SR function, cellular Ca stores or resting Ca concentration.

### Cardiac function in atrial-specific NCX1 KO mice

We assessed the cardiac function of 8–10 week old atrial-specific NCX1 KO mice using echocardiography. There was a trend but no significant reduction in ejection fraction or other parameters of LV function other than velocity of circumferential fiber shortening (Table 1). Left ventricular chamber size was mildly dilated with increased LV mass and wall thicknesses. Postmortem morphometric measures at age 8–10 weeks showed a significant increase of heart weight to body weight ratio in atrial-specific NCX1 KO mice compared to WT mice (WT: 4.58±0.14 mg/g, n = 32; KO: 6.67±0.18 mg/g, n = 35; *P*<0.001). This is a consequence of increased mass in both the atria and ventricles. The atrial weight to body weight ratio in WT was 0.22±0.02 mg/g (n = 18); in KO it was 0.40±0.02 mg/g (n = 21; *P*<0.001) and the KO atria were obviously dilated compared to WT. Notably we often found clots in the atria of KO animals despite heparinizing the mouse prior to thoracotomy, suggesting lack of normal atrial contraction. The ventricular weight to body weight ratio in WT was 4.04±0.20 mg/g, n = 18; in KO it was 5.68±0.13 mg/g, n = 21; p<0.001. We observed no significant gender differences (data not shown).

**Table 1:** Echocardiographic measurements of left ventricular dimensions and function in wildtype and atrial specific NCX1 knockout mice.

	Wildtype (n = 4)	Knockout (n = 4)
**Age, wk**	9.5±0.04	9.5±0.04
**Ventricular septal thickness, mm**	0.56±0.02	0.77±0.03 **
**Posterior wall thickness, mm**	0.52±0.03	0.77±0.09 *
**End diastolic dimension, mm**	3.58±0.23	4.80±0.25 **
**End systolic dimension, mm**	2.18±0.29	3.45±0.03 **
**Left ventricular fractional shortening, %**	39.96±4.76	27.59±3.46
**Velocity of circumferential fiber shortening, mm/s**	7.17±0.89	4.29±0.57 *
**Left ventricular ejection fraction, %**	75.78±4.63	59.63±5.34
**Left ventricular mass, mg**	58.80±7.17	145.79±10.88 ***

All values are means ± SEM.

* *P*<0.05, ** *P*<0.01 and *** *P*<0.001 as compared with WT.

### Whole organ electrophysiology

**Figure 3:**
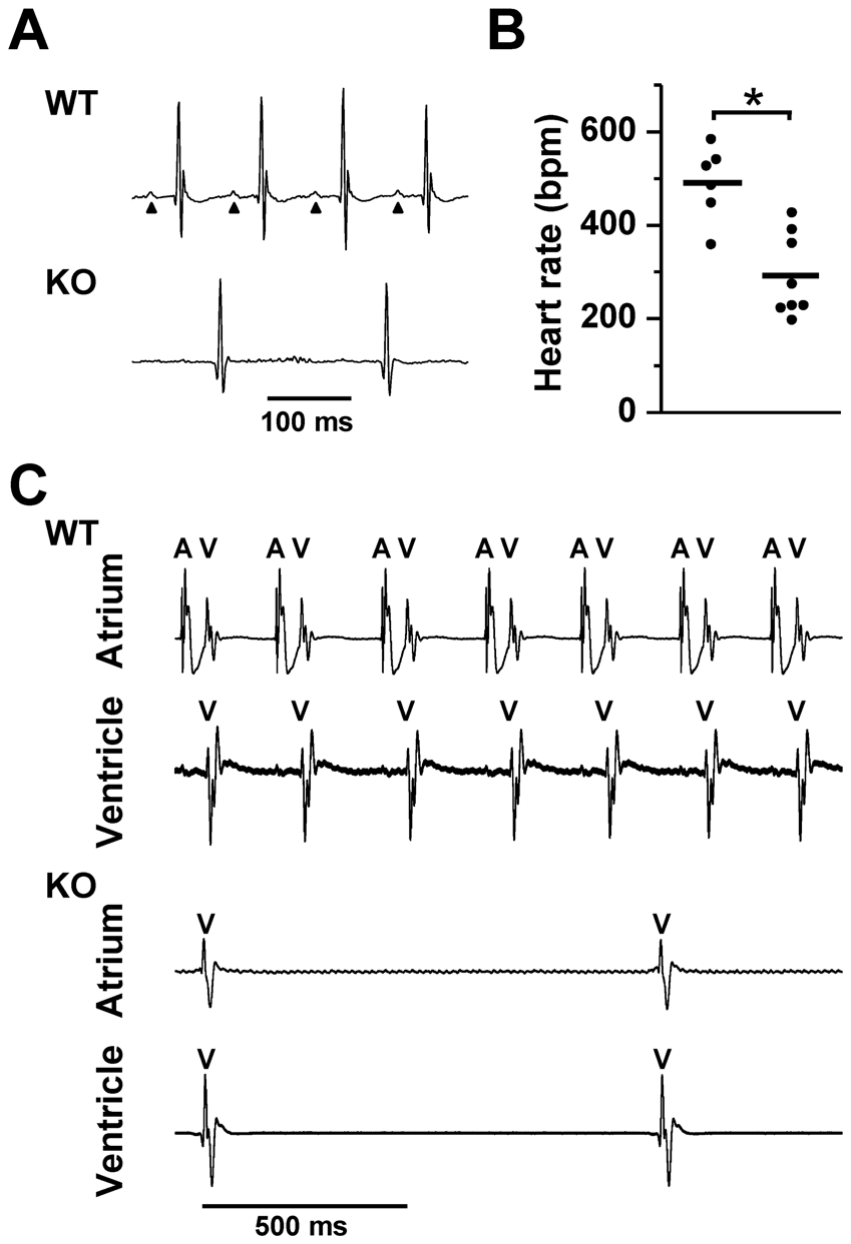
Sinus arrest and junctional rhythm in NCX1 KO mice. **A**. Representative telemetry ECGs from WT and atrial-specific NCX1 KO mice. WT mice were in normal sinus rhythm, with each P wave (arrows) followed by a typical murine QRS complex. In KO mice, P waves were conspicuously absent and a slow junctional escape rhythm (narrow QRS) was present. **B**. Mean ventricular (heart) rate in KO mice was lower than in WT mice (thick line, *P<0.001). **C**. Upper panels show simultaneous bipolar atrial and ventricular electrograms recorded from a representative WT heart. In the atrial lead, atrial (A) spikes are always followed closely (after physiologic delay) by a far-field ventricular (V) spike. The V spike is clearly shown in the ventricular lead below. The electrograms from a KO heart shown in the bottom panels exhibit only a V spike in both the atrial and ventricular leads indicating electrical silence of the atrium, consistent with the lack of P waves shown in **A**. There is no evidence of atrial fibrillation in the A lead of the KO heart.

During echocardiography, we noticed that the heart rate was slower in KO mice, which prompted us to perform electrocardiograms (ECGs) using an implanted telemetry system. ECGs from 8–10 week old WT mice showed normal sinus rhythm whereas those from KO mice lacked P-waves and had a slower ventricular rate (491±36 bpm in WT, n = 6 and 292±33 bpm in KO, n = 8; *P*<0.001; Figs. 3A and 3B). The morphology and duration of the QRS-complexes were similar in WT and KO mice suggesting that KO mice were in a junctional escape rhythm. To exclude the possibility that the slow ventricular response in KO mice was caused by underlying atrial fibrillation, we performed direct atrial and ventricular bipolar electrogram recordings in isolated Langendorff-perfused hearts from WT and KO mice (Fig. 3C). As expected in WT mice, atrial activity was followed, after a short physiologic delay, by ventricular activity. However in KO mice we were unable to detect atrial activity, suggesting atrial electromechanical standstill. The slower ventricular activity with short QRS duration and WT morphology in the KO hearts is consistent with a junctional escape rhythm originating in the His bundle region (Fig. 3C). We observed no other arrhythmias in the KO mice.

We subjected a subset of WT and atrial NCX1 KO mice to β-adrenergic (isoproterenol, ISO, 2 mg/kg IP) and muscarinic (carbachol, 1.25 mg/kg IP) stimulation (using saline controls). In response to ISO, the junctional escape rate in 3 NCX1 KO mice increased by 92% (from 252±7 to 483±5 bpm, *P*<0.01) compared to a 68% increase in sinus rate in 3 WT mice (411±4 to 691±5 bpm, *P*<0.01). In contrast, carbachol reduced the KO rate by 33% (224±6 to 151±3 bpm, *P*<0.01) compared to a 47% reduction in WT (410±5 to 218±4 bpm, P<0.01). Thus, atrial NCX1 KO mice were able to respond appropriately to adrenergic and muscarinic stimulation. However the underlying rhythm remained junctional.

### Whole atrium loaded with fluo-3-AM

**Figure 4:**
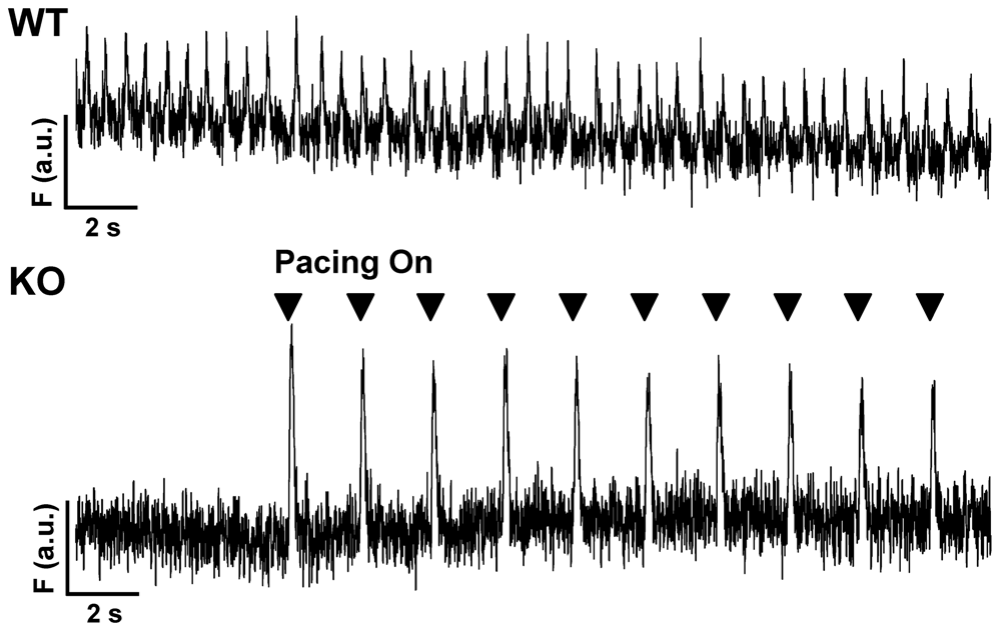
Fluo-3 transients recorded in a whole atrial tissue preparation. Fluorescence recordings from representative WT and KO whole atria (including SAN) loaded with the Ca indicator fluo-3 AM. The WT atria exhibited spontaneous fluo-3 Ca transients (*upper panel*). Ca transients were absent in the KO atria (*bottom panel*) until pacing was initiated at 0.5 Hz with external platinum electrodes embedded in the right atrium (Pacing On, markers indicate pacing).

We considered the possibility that the absence of atrial activity could result from conduction abnormalities in the remodeled atrial tissue. To address this question we separated the atria from the ventricles and loaded the entire atrial tissue preparation, which included the SAN, with fluo-3 by including 10 µM fluo-3 AM in the bath solution (30 min loading followed by three 10 min washes). We then monitored Ca transients at 22°C in the atrial tissue using a custom-made epifluorescence system. In WT (n = 3), we observed spontaneous Ca transients that were absent in KO (n = 3) until we applied external pacing from either the left or right atrium at 0.5 to 1 hz (Fig. 4). Thus atrial tissue from NCX1 KO mice is capable of depolarization and conduction, but this does not occur spontaneously. The result suggests that NCX1 KO mice have either defective impulse generation by the SAN or abnormal impulse propagation out of the SAN and into the surrounding atrial tissue. However the tissue itself can be stimulated electrically.

### Single SAN cell electrophysiology

**Figure 5:**
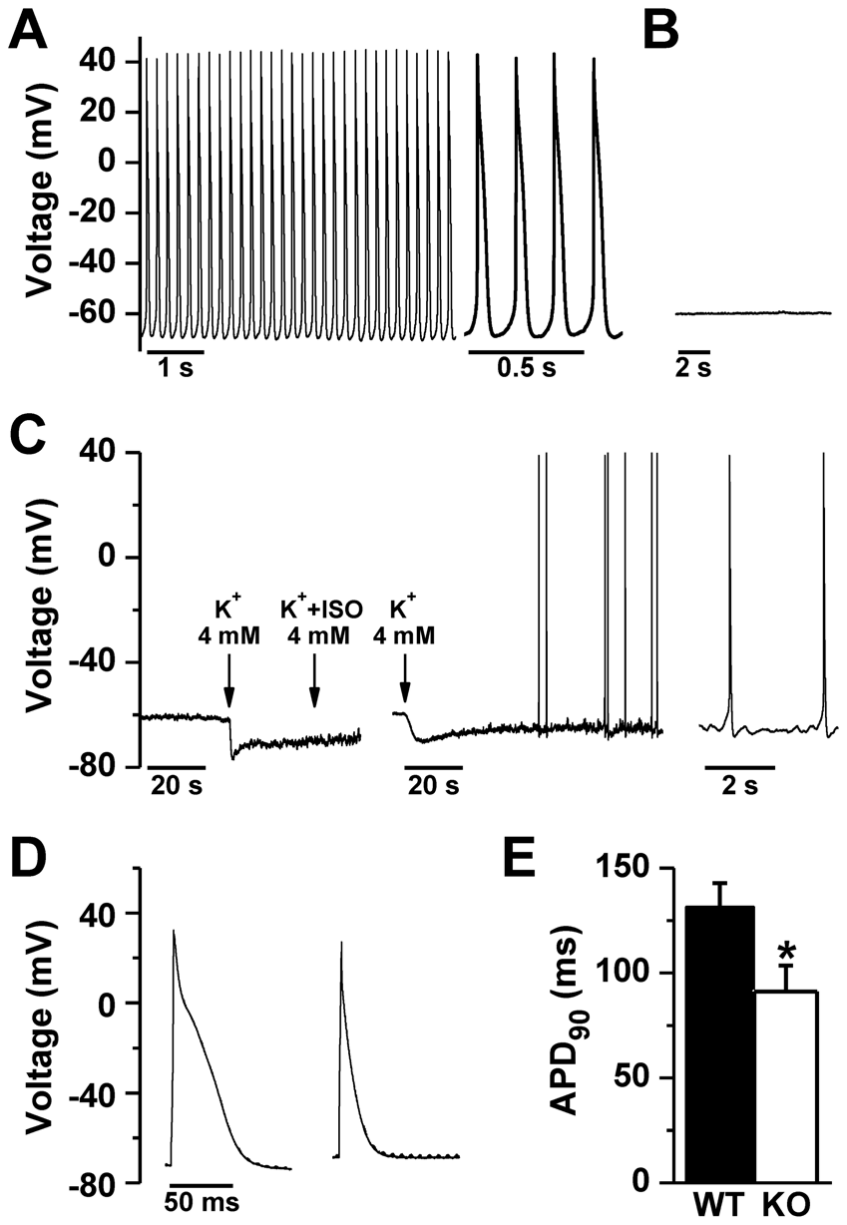
Action potentials in WT and NCX1 KO SAN cells. Spontaneous action potentials occurred at regular intervals in patch clamped WT SAN cells (**A**), but were absent in NCX1 KO SAN cells (**B**). **C**. In KO cells, lowering diastolic membrane potential by reducing bath K to 4 mM (arrow) sometimes elicited spontaneous APs. This only occurred 4 out of 7 times, even when isoproterenol (ISO) was added; two examples are shown, with the time scale expanded on the far right. **D**. WT (left) and quiescent NCX1 KO SAN cells (right) could be paced in current clamp mode. Note the reduced AP duration at 90% repolarization (APD_90_) compared to WT cells, summarized in panel **E** (*P<0.05 by t-test).

To explore the possibility of defective impulse generation by SAN cells, we used the whole cell patch clamp technique in current clamp mode to record membrane potential in isolated SAN cells from WT and NCX1 KO mice. In 30 out of 42 WT SAN cells, we detected spontaneous APs that lasted for at least 5 minutes (Fig. 5A). However, in KO SAN myocytes, we observed spontaneous APs in only 1 cell out of 27, and these were sparse and irregular. In the other 26 cells there was no spontaneous depolarization whatsoever (Fig. 5B). The maximum diastolic potential was more depolarized in KO SAN myocytes as compared to WT (KO: –58.3±1.8 mV, n = 23; WT: –69.6±2.6 mV, n = 18; *P*< 0.001). Since depolarization might suppress spontaneous APs by inactivating depolarizing currents, we hyperpolarized the resting membrane potential in 7 KO SAN cells by reducing K^+^ in the external solution from 5.4 to 4 mM. This maneuver initiated infrequent, but nonetheless spontaneous APs in 4 out of 7 KO SAN cells that were silent before hyperpolarization (Fig. 5C). Thus lowering the resting membrane potential did not restore normal pacemaker activity to KO SAN cells. Notably, we were able to pace quiescent KO SAN cells using current injection, indicating that these cells remain excitable although not spontaneously (Figs. 5D and 5E). The evoked AP was shorter in KO SAN myocytes as compared with WT (APD_90_: 131.3±11.5 ms in WT, n = 6, vs. 91.2±12.4 in KO, n = 4; *P*<0.05; APD_50_: 52.1±3.7 ms in WT vs. 27.9±3.4 in KO; *P*<0.01). Shorter APDs could be related to upregulation of outward currents as occur in ventricular NCX1 KO myocytes [18], but we did not examine this possibility. In some experiments we recorded AP-evoked fura-2 Ca transients. We found no significant difference in the amplitude of the AP-induced Ca transient between paced WT and paced KO SAN myocytes (WT: 349±95 nM, n = 6; KO: 456±124 nM, n = 4; *P* = 0.52). Similarly, there was no difference in the relaxation half-time (t_½_) of the Ca transient (WT: 82±5 ms; n = 6; KO: 82±7 ms, n = 4; *P* = 0.96).

### Funny current amplitude and kinetics in NCX1 KO SAN myocytes

**Figure 6:**
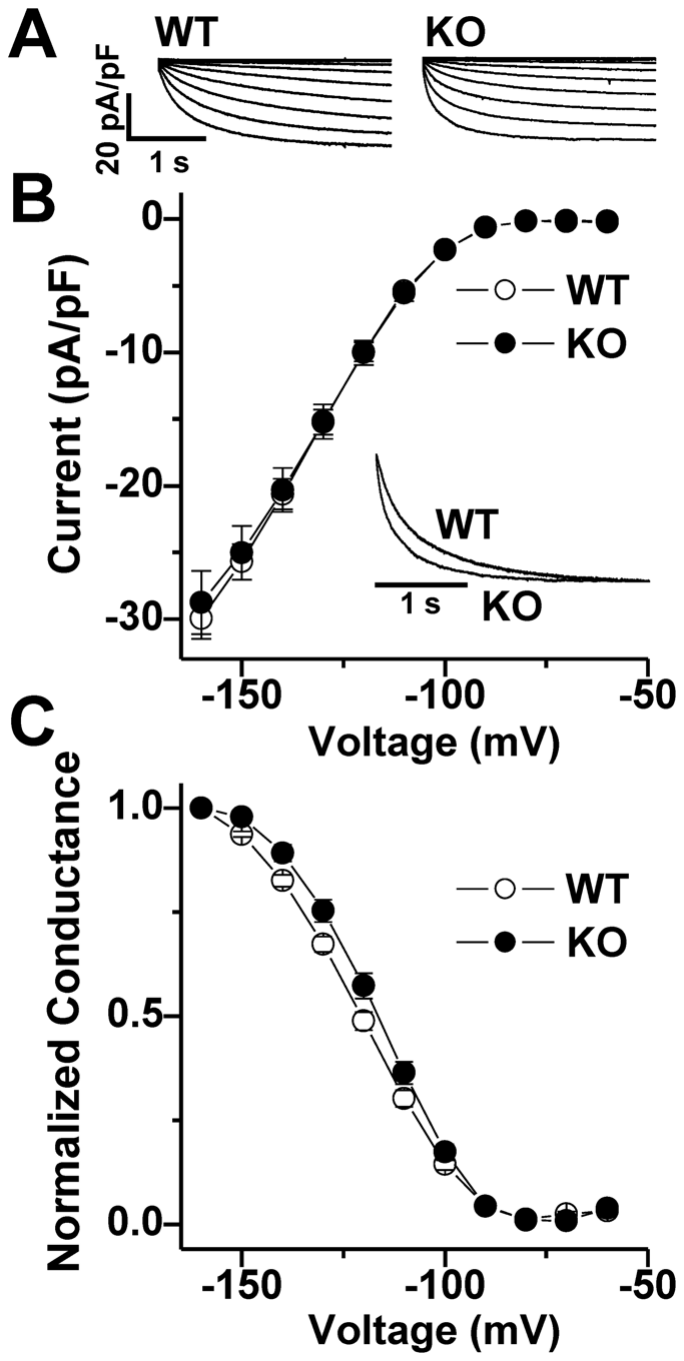
Funny current (I_f_) in WT and atrial-specific NCX1 KO SAN cells. **A**. Representative I_f_ current families (normalized to capacitance) in response to test clamps ranging from –60 to –160 mV in WT (left) and KO (right) SAN cells. **B**. There is no difference in the average peak current-voltage relationship for I_f_ recorded from 29 WT (open circles) and 40 KO SAN cells (closed circles). *Inset*: superimposed normalized current traces at –160 mV in a WT and KO SAN cell. **C**. Average conductance-voltage relationships for I_f_ in WT (open) and KO (filled) cells.

To determine whether maladaptive changes in I_f_ could contribute to the absence of spontaneous APs in NCX1 KO SAN cells, we recorded hyperpolarization-activated currents from isolated WT and KO SAN cells in the whole-cell patch-clamp configuration. We elicited I_f_ using 3 s hyperpolarizing voltage steps from –60 to –160 mV in 10 mV increments from a holding of potential of –50 mV to activate the current followed by a depolarizing step to +60 mV for 1s to deactivate current (Fig 6A). The I_f_ current density was indistinguishable in WT and KO cells at all potentials (Fig. 6B). However, double exponential fits of full-activated currents (at –160 mV; Fig. 6B inset) revealed accelerated activation kinetics for I_f_ in NCX1 KO cells compared to WT cells (KO at –160 mV: τ_fast_ =  126.2 ± 6.2 ms, n = 36; WT at –160 mV: τ_fast_ =  168.1 ± 11.1 ms, n = 29; *P*<0.01, Fig. 6B). To determine the voltage dependence of activation for I_f_, conductance (G) was calculated from hyperpolarization-activated inward currents and the resulting G-V relations were fit with a Boltzmann equation to yield midpoint activation voltages (V_½_) as previously described [19]. There was a small (∼4 mV) but significant depolarizing shift in the V_½_ for I_f_ in NCX1 KO compared to WT SAN cells (KO: V_½_ = –118.2 ± 1.3; WT –122.0 ± 1.2; *P*<0.05; Fig. 6C). We conclude that knocking out NCX has no effect on the maximum amplitude of I_f_, but there are subtle changes in activation properties that could potentially favor a greater role for I_f_ in KO cells.

### L-type Ca current amplitude and kinetics in NCX1 KO SAN myocytes

**Figure 7:**
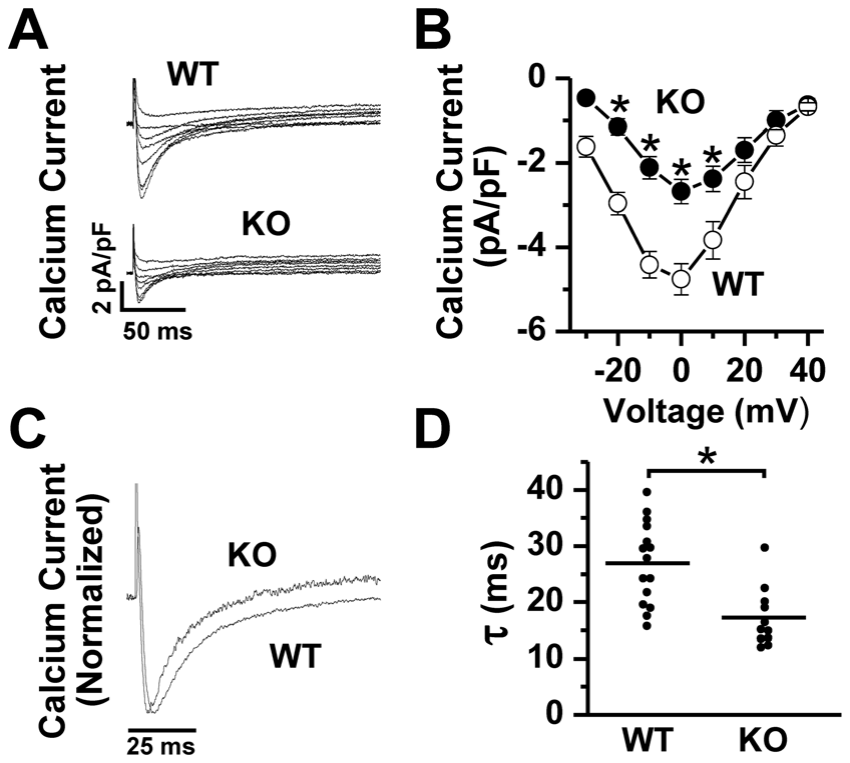
L-type calcium current in WT versus NCX1 KO SAN cells. **A**. Raw traces showing L-type Ca current (normalized to cell capacitance) recordings from representative whole cell patch clamped WT and KO SAN cells depolarized from a holding potential of –40 mV to test potentials ranging from –30 to +40 mV at 10 mV intervals. **B**. Mean current-voltage plots (± SEM) showing a significant decrease in peak Ca current in KO (*P<0.05 by post-hoc analysis of 2-way ANOVA). **C**. Representative current traces from WT and KO cells during a depolarization to 0 mV (normalized to peak) illustrating accelerated inactivation kinetics in NCX1 KO cells, as summarized in the plots shown in panel **D** (*P<0.05 by t-test).

We recorded whole cell L-type Ca current (I_Ca_) in WT and KO SAN cells. Cells were depolarized from –75 to –40 mV for 100 ms to inactivate Na^+^ current and then by 10 mV steps from –30 to +40 mV for 300 ms to activate I_Ca_. Despite the 2.5-fold increase in DHPR protein expression in KO (Fig. 1B), we observed a paradoxically reduced peak I_Ca_ amplitude at 0 mV in KO SAN myocytes compared to WT myocytes (KO: –2.7±0.3 pA/pF, n = 11; WT: –4.8±0.4 pA/pF, n = 15; *P*<0.001; Fig. 7A, 7B). Exponential fits of the decaying phase of I_Ca_ showed accelerated inactivation rate in NCX1 KO compared to WT SAN cells (KO: τ = 17.2±1.6 ms; WT: τ = 26.9±1.9 ms; *P*<0.001; Fig. 7C,7D). These changes in I_Ca_ amplitude and inactivation could be caused by increased subsarcolemmal Ca^2+^ resulting from the elimination of NCX, leading to increased Ca-dependent inactivation of the channels, similar to what we observed in ventricular-specific NCX1 KO myocytes [13], [20].

### Spontaneous calcium transients

The Ca clock model of pacemaker activity specifies that spontaneous local Ca release from the SR drives membrane depolarization by activating inward NCX current. The model predicts that in the absence of NCX there should be no spontaneous depolarization or APs. The fate of spontaneous Ca cycling in the absence of NCX is uncertain. Abrupt reduction of external Na has been shown to immediately eliminate Ca transients [7], but continued inhibition soon results in Ca waves caused by SR Ca overload. Bogdanov et al. [8] have shown that abrupt removal of NCX activity by Li substitution does not inhibit intracellular Ca cycling.

**Figure 8:**
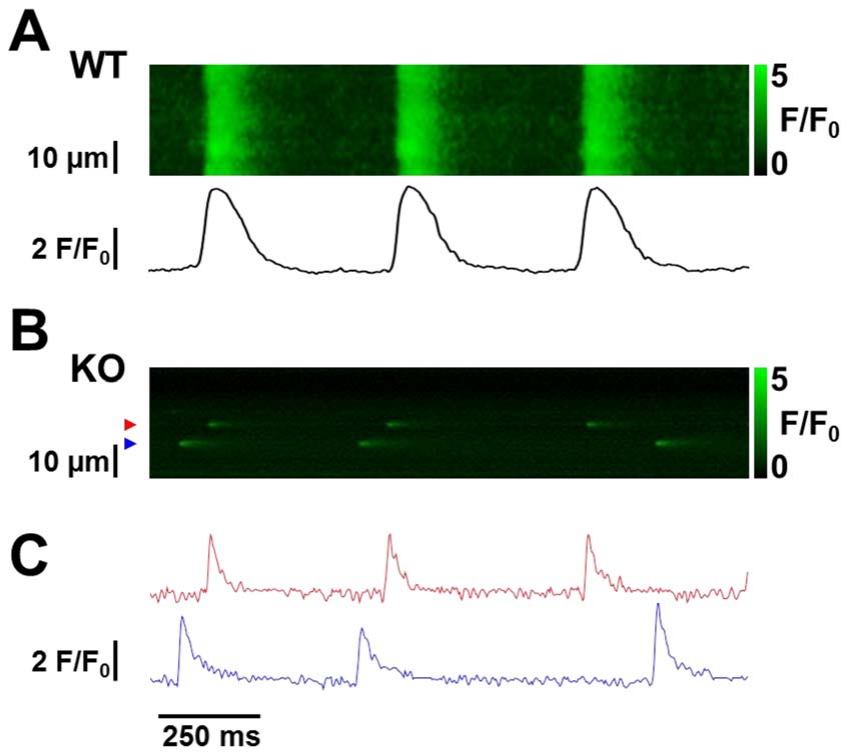
Confocal line scan images of fluo-4 loaded WT and NCX1 KO SAN cells. **A**. Typical line scan from a representative WT SAN cell showing periodic spontaneous Ca transients that activate with a rapid upstroke (spatial average shown directly below the image) consistent with depolarization. **B**. The line scan from a representative KO cell shows no Ca transients, but Ca sparks (i.e. local Ca releases, LCRs) do occur repetitively at specific locations. **C**. Fluorescence traces for the locations corresponding to the two sparks shown in panel **B** identified using color-coded arrows**.**

To address this issue, we performed line scan images of spontaneous Ca release in non-paced fluo-4 AM loaded SAN cells isolated from WT and NCX1 KO mice (Fig. 8). In WT mice, we routinely observed regular spontaneous synchronous Ca release consistent with depolarization (Fig. 8A). In KO cells, we rarely observed synchronous whole cell Ca release. Instead in all 37 KO cells studied we always observed Ca sparks, i.e. local Ca release events (LCRs), repeatedly firing at fixed locations over the course of the recording (Fig. 8B). In the Ca clock model, LCRs induce inward NCX current leading to accelerated depolarization and ultimately an AP. Thus, we find that knockout of NCX1 is associated with failure of AP-induced spontaneous Ca transients, but not LCRs, supporting the role of NCX as the critical link between Ca release and membrane depolarization.

## Discussion

The role of NCX as a critical participant in the genesis of cardiac SAN pacemaker activity through either a Ca-clock or a Coupled-clock mechanism is controversial [4]. This concept departs from the prevailing hypothesis of pacemaker function for the last 20 years, which has revolved around the so-called “funny current” (I_f_) through hyperpolarization-activated cyclic nucleotide-sensitive (HCN) channels [2]. To address these hypotheses, we generated atrial-specific NCX1 KO mice that live into adulthood despite complete absence of NCX1 in the atrium and SAN. Our major finding is that these mice exhibit no evidence of atrial depolarization on ECG and instead manifest a junctional escape rhythm. Furthermore, isolated SAN cells from the NCX1 KO mice are devoid of spontaneous APs, despite the continued presence of I_f_ and normal intracellular Ca stores. These results strongly support the hypothesis that NCX and the Ca clock are critical elements of the SAN pacemaker mechanism.

Previous studies have evaluated the role of NCX on spontaneous pacemaker activity in the SAN. Bogdanov et al. [8] showed that substituting Li for Na resulted in SAN arrest. However, Na replacement affects other ion channels (including I_f_) complicating interpretation. Application of KBR7943 to block NCX also suppresses spontaneous beating in SA nodal cells [7]. Unfortunately, KBR7943 is a very non-specific blocker of NCX1 [21]. Gao et al. [10] showed that partial ablation of NCX1 (70–80% knockdown) using an αMHC-inducible Cre transgenic line known to show mosaic deletion in the atria [22], has little effect on the baseline rate of spontaneous APs in the SAN. This suggests that even a small amount of NCX1 can provide sufficient depolarizing current to maintain SAN activity. This contrasts with our model of complete knockout of NCX1 using the SLN-Cre knockin line where we have no SAN activity whatsoever. However it is difficult to explain why the incomplete NCX1 KO of Gao et al. had a blunted chronotropic response to β-adrenergic stimulation. Since I_f_ was intact, abnormal Ca handling in the face of reduced NCX may have been responsible, particularly since I_Ca_ was not reduced. Unfortunately SR Ca content was not reported. In our complete KO of NCX1, we observed an appropriate chronotropic response to the β-agonist isoproterenol, though this was in the setting of a baseline junctional rhythm.

Herrmann et al. also created a partial NCX1 KO model in SAN cells (∼90%) by using an inducible and HCN4-specific Cre line [9]. Similar to our results, they described ventricular enlargement. However they found inconsistent disruption of normal sinus rhythm on ECG and telemetry along with an unexplained increase in ventricular arrhythmias, possibly due to the undetected presence of HCN4 in conduction tissue in the ventricles. Furthermore, it is not certain whether I_f_ or NCX current was affected in their model as membrane currents were not assessed. Thus it is not possible to determine from their data the actual cause of disrupted sinus rhythm in their mice.

Unlike the pharmacologic and genetic manipulations described above, the total ablation of NCX1 in the atria and SAN of our genetically modified mice avoids potential off-target effects of drugs as well as complications of incomplete or off-target knockout of NCX1. It is in fact critical to be certain that the entire SAN is knocked out for any experiments examining the importance of NCX in SAN pacemaking. Gene painting for example [10], could exclude regions of the SAN from KO. In our approach, adaptations in protein expression and chamber remodeling must be considered. Nevertheless, we found no major changes in the electrophysiologic properties of I_f_ in isolated NCX1 KO SAN cells (Fig. 6). Thus we cannot attribute the lack of spontaneous SAN beating to failure of I_f_. If anything, the minor changes in activation properties and V_½_ of I_f_ would be expected to increase the likelihood of spontaneous depolarization. We also found that spontaneous Ca release in the form of recurring LCRs persisted after ablation of NCX1 (Fig. 8) despite adaptations of other Ca regulatory proteins, which supports the concept that NCX is required to couple Ca release by the SR to depolarization of the membrane.

Spontaneous AP firing rate in the SAN is correlated with cAMP-dependent phosphorylation of critical proteins involved in Ca handling, such as I_Ca_ and the SERCA regulatory protein phospholamban [23]. It has also been demonstrated that Ca-stimulated adenylate cyclases in SAN cells are important regulators of I_f_ and other PKA dependent targets including PLB [24], [25], [26]. We have no indication that NCX1 KO SAN cells have a reduced phosphorylation status or reduced PKA or cAMP that might explain lack of pacemaker activity. On the contrary, we would speculate that there is an increase in Ca-dependent phosphorylation of PLB and other proteins given the high likelihood that subsarcolemmal Ca is increased in the absence of NCX (as we have observed in ventricular NCX1 KO cells [20]).

We found that atrial NCX1 KO mice have increased heart weight to body weight ratio. All four cardiac chambers are enlarged, including the ventricles where there is no change in NCX1 protein expression. The increase in chamber size is most likely an adaptation to the slow native heart rate of the KO mice. A similar increase in chamber sizes occurs in models of induced complete heart block where heart rates are inherently slow and cardiac output depends upon increases in stroke volume provided by chamber dilation and hypertrophy [27], [28], [29]. We considered the possibility that remodeling of the SAN and atria in the NCX1 KO could impede impulse conduction across the tissue. However, we were able to pace the atrial tissue preparation (Fig. 4), which indicates that electrical connectivity is intact despite remodeling.

We surmise that the escape rhythm of atrial-specific NCX1 KO mice originates in the His bundle region since the QRS duration and morphology in the KO are similar to the QRS duration and morphology in the WT. This indicates that the activation order in the two groups is through the normal His-Purkinje-Ventricular sequence. Since we did not observe any evidence of spontaneous depolarization in the atria (no P waves by electrocardiogram, and no Ca transients in the isolated atrial tissue), it seems unlikely that the escape rhythm could originate in a subsidiary pacemaker located in the atrium, as has been described in cats [30] and dogs [31]. It is also noteworthy that we did not observe any retrograde conduction from the ventricles into the atria (Fig. 3). The reason for this is unclear, but speculatively could be due to compensatory reductions in AV node I_Ca_, which is thought to be essential for AV node conduction [32], [33], [34]. However, we cannot exclude the possibility that a conduction abnormality in the atrial tissue is sufficient to block retrograde impulses from the AV node.

Despite an increase in DHPR expression, we found a ∼50% reduction in I_Ca_ in NCX1 KO SAN cells (Fig. 7). The reasons for this difference are not clear, but could be related to differences in membrane targeting in the KO. I_Ca_ is the major depolarizing current for Phase 0 of the action potential in SAN cells, and thus a critical aspect of both the M clock and Ca clock models, and by extension the Coupled clock model. In addition, I_Ca_ provides the Ca that refills the SR Ca stores under the Ca and Coupled clock models [4]. Ca channel blockers and genetic ablation of the L-type Ca channel (Ca_v_1.3) interfere with pacemaker activity in the SAN [35], [36], [37]. Thus we cannot exclude the possibility that the reduction in I_Ca_ contributed to the suppression of APs in our KO mice. However, in our atrial NCX1 KO mice reduction of I_Ca_ does not lead to failure of LCRs that repeat at specific sites in quiescent cells (Fig. 8). Furthermore the reduced I_Ca_ in the KO maintains SR Ca load when the cells are paced (Fig. 6), though this prepulse pacing never produced Ca waves. Thus the refilling of SR Ca stores by I_Ca_, the hypothesized role of this current in the Ca clock mechanism [4], remains intact. It is possible that the SR Ca load of quiescent KO cells is decreased compared to WT cells that are beating spontaneously. However, the persistence of LCRs in the KO indicates that any reduction in spontaneous APs is a consequence of NCX ablation rather than depletion of SR Ca.

We also found that there was a slight depolarization of the maximum diastolic potential in KO cells, which could partially inactivate I_f_ and thus pacemaker activity despite the preserved I_f_ properties observed in our voltage clamp experiments. However, even after we lowered the membrane potential by reducing extracellular K, we rarely observed spontaneous APs, and these were both infrequent and irregular. Thus, while it is unlikely that the slight depolarization is a major factor blunting pacemaker activity, it could be contributory.

It is curious that SAN cells adapt to the absence of NCX by increasing PMCA and decreasing SERCA (Fig. 1). These two adaptations should have contradictory effects, with the former increasing Ca removal from the cytosol, and the latter decreasing it. The expectation would be a decrease in SR Ca load, and possibly an increase in resting Ca. However, we found no evidence of altered Ca homeostasis. Diastolic Ca is not elevated in KO mice, and SR load, release and uptake are intact as shown by fura-2 measurements of caffeine-induced SR Ca release (Fig. 2). We observed a similar reduction in SERCA but with preserved SR stores and Ca cycling in heart tubes isolated from global NCX KO embryos at day 9.5 post coitum [38]. Possibly, phospholamban regulation of residual SERCA protein allows for normal Ca uptake by the SR. Alternatively, it has been shown that dramatic decreases in SERCA activity have surprisingly minimal effects on SR Ca content in ventricular cells [39]. This is because of the steep dependence of SR Ca release on SR Ca content, which has been demonstrated by several groups (recently summarized by Eisner et al. [40]). In the special case of NCX1 KO mice, there is no NCX to compete with SERCA for reuptake of released Ca. Thus most of the Ca released from the SR in KO cells will cycle back into the SR except for the small amount pumped out through PMCA, and replenished by I_Ca_. Mitochondria have also been shown to influence Ca cycling in SAN cells, however the effects on pacemaker rate are modest [41] and likely operate through competition with SR for Ca. We have no direct information on mitochondrial Ca uptake in our NCX1 KO cells, but we do know that SR Ca is not depleted and is thus not a likely cause of impaired pacemaker activity.

We found that NCX1 KO SAN cells continue to exhibit LCRs that repeat at the same location but have no Ca transients indicative of depolarization (Fig. 8). Furthermore, we did not observe spontaneous Ca waves, even after applying prepulses in patch clamped cells. Sanders et al. [7] and Bogdanov et al. [8] both observed persistent Ca waves after blocking NCX using Li substitution for external Na. We found that genetic ablation of NCX1 resulted in abolition of Ca transients, but not repetitive LCRs. The persistent LCRs we observed (Fig. 8) suggest that there is continued cycling of intracellular Ca despite the absence of NCX1. However we are uncertain as to why LCRs failed to ignite Ca waves. The implication is that LCRs are not sufficient in terms of spatial distribution or amplitude to trigger a critical number of adjacent Ca release units to generate waves. Possibly Ca buffering by fluo-4 confines LCRs spatially. Nevertheless it is not surprising that LCRs are unable to depolarize the membrane to generate APs in the absence of NCX1.

In conclusion, we find that I_f_ (M clock) is not sufficient to spontaneously depolarize SAN cells in the complete absence of NCX1 conferred by genetic modification. This result unequivocally demonstrates the essential role of NCX and cellular Ca cycling in normal pacemaker activity, and could be used to develop new approaches to treating sinus node disease.

## Methods

### Generation of atrial-specific NCX KO mice

We produced atrial-specific NCX1 KO mice using Cre/loxP technology. We crossed mice that were hemizygous for Cre recombinase expression under the control of the endogenous SLN promoter [12] with our previously derived NCX1 exon 11 floxed mice [13]. In the heart, SLN is expressed exclusively in the atrium [42]. Cre-expressing offspring were viable and lived into adulthood. The animals used in this study were between 8 and 13 weeks of age.

### Echocardiography

Mice were sedated with isoflurane vaporized in oxygen and ultrasonically imaged with a Siemens Acuson Sequoia C256 instrument (Siemens Medical Solutions, Mountain View CA) as previously described [13], [43]. 2-D guided M-mode images were analyzed for left ventricular cavity dimensions (end diastolic dimension - EDD and end systolic dimension - ESD) and wall thickness (posterior wall thickness - PWT and ventricular septal thickness - VST) during systole and diastole. Ejection times and heart rates were determined from Doppler images. Left ventricular mass was calculated from the EDD, PWT and VST values according to Tanaka et al [44]. Left ventricular function was determined from three measures: fractional shortening (%LVFS); velocity of circumferential fiber shortening (VCF) and ejection fraction (EF).

### Electrocardiograms

Mouse ECGs were obtained using implantable telemetry (TA10ETA-F20 or TA10 ETA-F10; Data Sciences Intl., St. Paul, MN) as we have described previously [43]. In addition to baseline recordings, ECG telemetry data were obtained continuously just prior to and for up to 4 hours after ISO (2 mg/kg IP), carbachol (1.25 mg/kg IP) and saline control injections. Data waveforms and heart rate parameters were analyzed with the Data Sciences Intl. analysis programs (ART 4.1).

### Cardiac electrograms in Langendorff-perfused hearts

We perfused the aorta in a retrograde fashion at a rate of 3 ml/min and at 36.5°C with oxygenated Tyrode solution containing (in mM): 136 NaCl, 5.4 KCl, 10 HEPES, 1 MgCl_2_, 0.33 NaH_2_PO_4_, 1.8 CaCl_2_, 10 glucose; pH adjusted to 7.4 with NaOH. We positioned two widely spaced electrodes on the right atrium and the left ventricle to record a “pseudo-electrocardiogram,” as previously described [45].

### Isolation of sinoatrial myocytes from adult mouse hearts

We removed hearts via thoracotomy from heparinized (300U IP) mice anesthetized with isoflurane. We then separated the atria from the ventricles and dissected the SAN node region at 37°C in heparinized (10 U/ml) Tyrodes solution, which consisted of (in mM) 140 NaCl, 5.4 KCl, 1.2 KH_2_PO_4_, 5 HEPES, 5.55 glucose, 1 MgCl_2_, 1.8 CaCl_2_; pH adjusted to 7.4 with NaOH. We identified the SAN node region by the borders of the superior and inferior vena cavae, the crista terminalis and the interatrial septum [35], [46]. Nodal tissue was digested by collagenase type II (Worthington Biochemical), protease type XIV (Sigma-Aldrich), and elastase (Worthington Biochemical) for 30–35 min at 37°C in a modified Tyrodes solution containing (in mM): 140 NaCl, 5.4 KCl, 1.2 KH_2_PO_4_, 5 HEPES, 50 taurine, 1 mg/ml BSA, 18.5 glucose, 0.066 CaCl_2_; pH adjusted to 6.9 with NaOH. After digestion, tissue was transferred to a modified Kraft-Bruhe (KB) solution containing (in mM): 100 K- glutamate, 10 K- aspartate, 25 KCl, 10 KH_2_PO_4_, 2 MgSO_4_, 20 taurine, 5 creatine, 0.5 EGTA, 20 glucose, 5 HEPES, and 1.0% BSA; pH adjusted to 7.2 with KOH at 37°C. Cells were dissociated by pipetting for 10 min with a wide mouth fire-polished glass pipette. After gradual reintroduction of Ca to a final concentration of 1.4 mM, dissociated cells were stored at room temperature for up to 6 h until recording.

### Immunochemistry

We plated isolated SAN myocytes on 0.01% poly-L lysine coated coverslips. Cells were allowed to adhere to the coverslips for ∼2 hours before fixation with 4% paraformaldehyde for 20 min. We then permeabilized the myocytes with 0.02% Triton-X 100 in PBS and blocked non-specific immunoreactive sites with 10% normal goat serum in PBS. When mouse secondary antibodies were used, the samples were also pre-incubated with unconjugated goat-anti-mouse secondary antibodies for 1 h at room temperature. After washing 4x for 5 min with PBS, samples were incubated with primary antibodies overnight at 4°C. They were then washed in PBS and incubated with fluorescently labeled secondary antibodies for 1 h at room temperature. Coverslips were mounted onto slides with Prolong Gold (Invitrogen), which was allowed to dry 24 hours before imaging.

### Antibodies

Primary antibodies used for immunofluorescence were rabbit anti-HCN4 (Alomone APC-052, 1∶200) and mouse anti-NCX (R3F1, [47], 1∶100). Secondary antibodies used were goat anti-rabbit Alexa 488 or goat anti-mouse Alexa 568 (Invitrogen A-11034 and A-11031, 1:1000). For immunoblots, primary monoclonal antibodies were anti-NCX1 (R3F1) or anti-PMCA (MA3-914), anti-SERCA (MA3-919), or anti-α2DHPR (MA3-921) from Thermo Scientific. Antibodies were detected by direct conjugation to horseradish peroxidase (AbD Serotec).

### Imaging in Fixed Cells

Immunofluorescence images of isolated SAN cells were collected with an Olympus IX81 inverted spinning disc confocal microscope using a TIRFM PLAN APO 60x 1.45 N.A. objective (Olympus Imaging America, Inc., Center Valley, PA). For each experiment, we always processed one NCX1 KO mouse and one WT littermate in parallel. Negative controls were conducted where the primary antibody was removed or substituted with IgG isotype control antibodies. For cells using the HCN4 antibody, negative controls were conducted using an epitope blocking peptide (Alomone Laboratories). For each pair of samples, image acquisition settings were identical. Channels in all images were normalized to the maximum pixel intensity of the WT samples using ImageJ v1.46 software [48]. After normalization, the bottom 10% of pixels were removed (intensity <25; scale 1 to 255), and the normalized mean pixel intensity was calculated from a region of interest (ROI) that included only the tissue or cell sample.

### Single Cell Electrophysiology

For whole-cell current recordings, we placed the cells in an experimental chamber (0.25 ml) mounted on the stage of a Nikon Diaphot inverted microscope modified for simultaneous electrophysiology and fluorescence recording. We voltage clamped myocytes using an Axopatch 200B patch clamp amplifier (Molecular Devices, Sunnyvale, CA) under the control of pClamp 9 software (Molecular Devices) and a Digidata 1322A PC interface (Molecular Devices). We applied series resistance compensation to all voltage clamp recordings. A series of five 100 ms conditioning pulses at 1 Hz from –75 to 0 mV preceded every recording of fura-2 loaded cells to stabilize SR Ca load. For I_NCX_ measurements the bath was a modified Tyrodes solution containing (in mM): 136 NaCl, 5.4 KCl, 10 HEPES, 1 MgCl_2_, 0.33 NaH_2_PO_4_, 1.8 CaCl_2_, 10 glucose; pH adjusted to 7.4 with NaOH. For I_Ca_ measurements, the bath solution contained (in mM): 130 NaCl, 5.4 CsCl, 10 tetraethylammonium Cl, 0.33 NaH_2_PO_4_, 10 HEPES, 1 MgCl_2_, 1.8 CaCl_2_, 10 glucose; pH adjusted to 7.4 with CsOH. The pipette solution for measuring I_Ca_ and I_NCX_ contained (in mM): 130 CsCl, 10 tetraethylammonium Cl, 10 NaCl, 10 HEPES, 5 Mg-ATP, 5 phosphocreatine, 0.05 cAMP; pH adjusted to 7.2 with CsOH. To measure hyperpolarization-activated I_f_ currents, we used methods described by Liao et al [19]. The pipette solution contained (in mM): 128 K- aspartate, 7 KCl, 1 MgCl_2_, 10 HEPES, 1 CaCl_2_, 10 EGTA, 4 Mg-ATP, 6.6 Na-phosphocreatine, 0.1 Na-GTP (pH = 7.2 with KOH). The bath solution contained (in mM): 140 NaCl, 5.4 KCl, 10 HEPES, 1 MgCl_2_, 1.8 CaCl_2_, 10 glucose, 1 BaCl_2_ (pH 7.4 with NaOH). Conductance (G) for I_f_ was determined from inward hyperpolarization-activated currents according to the equation G = I/(V-V_rev_), where V_rev_ for I_f_ was taken as -30 mV [19], [46] and I_f_ was measured as the fully activated steady-state current achieved at each hyperpolarizing voltage. Voltages in I_f_ recordings were corrected offline to compensate for a 17 mV junction potential error (calculated using pClamp 9 junction potential calculator, and based on the solutions and temperature used for these experiments).

We measured spontaneous and induced action potentials (APs) with the current clamp mode of the whole cell patch clamp technique. We used the I_NCX_ bath solution described above but lowered the CaCl_2_ concentration to 1 mM. The internal solution contained (in mM): 130 KCl, 10 NaCl, 10 HEPES, 0.2 EGTA, 2 Mg-ATP, 6.6 phosphocreatine, 0.05 cAMP, 0.06 free Ca; pH adjusted to 7.2 with KOH.

### Fluorescence measurements

We recorded fura-2 fluorescence signals during voltage and current clamp using a custom-designed photometric epifluorescence detection system. The inverted microscope used for patching (Nikon Diaphot) was modified for dual wavelength excitation at 360 and 400 nm from LED light sources using an electronic chopper [49]. All fura-2 recordings were performed at 34°C. The fluorescence emission was measured at 510±40 nm as described in detail previously [15]. Fluorescent emission measurements corresponding to these two excitation wavelengths were simultaneously recorded using the Axopatch 1322A digitizer. Cells were loaded with the Ca indicator fura-2 by incubating cells in standard bath tyrodes containing fura-2 AM (10 µM) and Pluronic F-127 (0.02%) for 30 minutes, followed by 3 washes for 5 minutes each. Ca concentration was calculated from the ratio (R) of the fluorescence intensities at the 2 excitation wavelengths (ratios at 700 Hz) using the method of Grynkiewicz et al. [50] according to the equation:

[Ca^2+^]_i_ = K_d_ x (*S*_f2_/*S*_b2_) x ((R-R_min_)/(R_max_-R)),

where K_d_ is the dissociation constant of fura 2 (224 nM), R is the fluorescence ratio obtained at 360 nm/400 nm, R_min_ and R_max_ are the ratios at zero and saturating Ca, and *S*_f2_/*S*_b2_ is the ratio of zero Ca over saturating Ca fluorescence intensities at 400 nm.

For whole atria with intact SAN, we recorded fluo-3 fluorescence signals using a similar custom-made system, except that the excitation LED wavelength was 488 nm, and the emission wavelength was >510 nm.

### Statistical analysis

Data are expressed as mean ± S.E.M where applicable. Error bars are shown only if larger than symbols. Student's *t* test was used for direct comparisons of WT *versus* KO. 2-way ANOVA was used for comparison of voltage dependence. A *P* value of <0.05 was considered significant.

### Study Approval

This study was carried out in strict accordance with the recommendations in the Guide for the Care and Use of Laboratory Animals of the National Institutes of Health. All mouse experiments were approved by the Institutional Animal Care and Use Committee at Cedars-Sinai Medical Center (IACUC #: 003574) and the Chancellor’s Animal Research Committee at UCLA (Protocol #: 1992-263-53). We anesthetized the mice with isoflurane prior to heart removal, and all efforts were made to minimize suffering.
